# A Systematic Review and Meta-Analysis of Basal and Post-Stress Circulating Cortisol Concentration in an Important Marine Aquaculture Fish Species, European Sea Bass, *Dicentrarchus labrax*

**DOI:** 10.3390/ani13081340

**Published:** 2023-04-13

**Authors:** Athanasios Samaras

**Affiliations:** Independent Researcher, 71303 Heraklion, Greece; samaras.thanas@gmail.com

**Keywords:** aquaculture, cortisol, European sea bass, meta-analysis, reference values, stress, welfare

## Abstract

**Simple Summary:**

European sea bass is a species with high economic and societal value in the Mediterranean due to its intensive use in aquaculture. However, it is a species characterized by high cortisol levels that show high variation. The present systematic review and meta-analysis collected and examined all the published data on circulating cortisol in this species. The aim of the study was to analyze all published values in order to provide normal values and ranges of plasma cortisol in this species, both in basal and post-acute stress conditions. Results revealed a very high between-study heterogeneity, while it also calculated the pooled levels of cortisol and their confidence intervals for both basal and post-stress conditions. Moreover, results were analyzed based on various parameters that can potentially affect cortisol levels, including technical, such as assay type and rearing unit, as well as biological, such as body size and anesthesia, influences.

**Abstract:**

Background: European sea bass is a species characterized by high and dispersed cortisol levels. The aim of the present study was to analyze all published data on basal and post-acute stress cortisol levels in this species. Methods: For this systematic review and meta-analysis the Web of Science and Scopus databases were searched for papers reporting plasma or serum cortisol levels in E. sea bass, without language or date restrictions. Data were extracted directly for the reported results and were analyzed separately for basal and post-acute stress levels, as well their standardized mean differences (SMD) using random-effects meta-analyses. Results: Of 407 unique records identified, 69 were eligible. Basal cortisol levels had a pooled effect of 88.7 ng mL^−1^ (*n* = 57), while post-acute stress levels were 385.9 ng mL^−1^ (*n* = 34). The average SMD between basal and post-stress was calculated to be 3.02 (*n* = 22). All analyses had a high between-study heterogeneity. Results for basal and post-stress levels were affected by the assay type and anesthesia prior to blood sampling. Conclusions: Cortisol levels in E. sea bass are higher than most studied fish species and display large heterogeneity. Application of stress led to elevated cortisol levels in all studies examined. In all cases, sources of between-studies heterogeneity were identified.

## 1. Introduction

Cortisol is the major stress hormone in fish [1,2,3]. It is the final product of the action of the Hypothalamus–Pituitary–Interrenal tissue (HPI) axis, and it has been reported to respond with increased concentrations to various types of stress in order to regulate stress responses [1,2,3]. Apart from controlling the stress responses, cortisol is a regulatory hormone for both metabolism and osmoregulation in fish [3]. Therefore, it is a crucial hormone in the physiology and biology of fish.

European sea bass, *Dicentrarchus labrax*, is a fish species with high economic value due to the fact that it is one of the main marine aquaculture fish species in the Mediterranean. Although widely cultured, this species shows high cortisol responses to stress compared to other species widely cultivated in the Mediterranean, such as gilthead seabream, *Sparus aurata*, and meage, *Argyrosomus regius* [4,5], as well as high variation in cortisol concentrations in basal (pre-stress) and post-stress conditions [6]. A decade ago, Ellis et al. (2012) [6] reported that a high between-study variation in the basal and post-stress cortisol concentration can be observed in this species, proposing five possible sources of variation. Recent research has shown that most of these sources indeed can add variation, while other factors remain untested still (Table 1).

### 1.1. Rationale for Meta-Analysis

As discussed earlier, cortisol variation in E. sea bass is large, both in the same study (within population [8]) and between different studies. This makes the generalization of conclusions based on a single measurement impossible, since under such conditions it is hard to define accurate reference values. However, cortisol measurement is an important indicator of the physiological status of fish in terms of stress, osmoregulatory, and metabolic regulations [2]. A meta-analysis of data from different independent studies can provide a quantitative statistical way to combine their results. Especially in cases with data that show high heterogeneity, such as cortisol in E. sea bass, a meta-analysis using random effects models allows for important conclusions to be drawn. Moreover, certain meta-analysis statistical tools provide the means to incorporate other effects, such as environmental (husbandry, water quality etc.), biological (body weight), and technical (method used to quantify cortisol), in the analysis. For instance, the assay type used to measure cortisol in each study (ELISA, RIA, HPLC) can be included in a sub-group meta-analysis to provide information on whether the assay type affects the outcome of the study.

### 1.2. Objectives

The objective of the present study was to provide normal values and ranges of plasma cortisol in E. sea bass, both in basal (i.e., without experimental exposure to any stressors) and post-acute stress conditions, as well as quantify the standardized mean difference between basal and post- stress (both acute and chronic) cortisol levels. Moreover, this study aimed at investigating the effects of (1) cortisol measurement assay type, (2) type of rearing unit, (3) use of anesthesia during the blood sampling, (4) water parameters such as temperature, salinity, dissolved oxygen, pH, (5) fish body weight, (6) stocking density, and, in the cases of post-stress levels, (7) the time after stress that the blood sampling took place.

## 2. Materials and Methods

The Preferred Reporting Items for Systematic reviews and Meta-Analyses Statement (PRISMA) guidelines to plan, implement, and report this systematic review and meta-analysis have been followed in this study [25]. The PRISMA checklist is available in Supplementary Table S1.

### 2.1. Identification of Studies

**Figure 1:**
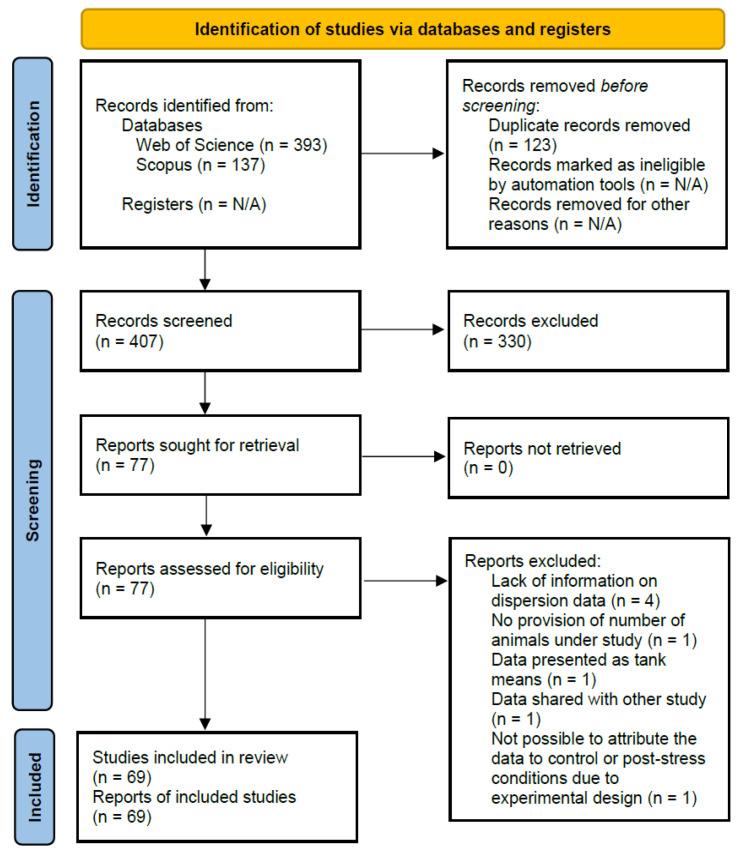
Flow diagram showing phases of systematic review and study selection for meta-analysis.

The databases Web of Science and Scopus were assessed using the search terms (“European sea bass” OR “sea bass” OR dicentrarchus OR labrax) AND (cortisol OR glucocorticoid OR corticosteroid) to find peer-reviewed articles reporting cortisol levels in E. sea bass, until the date of search (7 March 2022). Figure 1 shows a flow diagram that summarizes all stages of the systematic review process, including the numbers of studies identified at each stage and any reasons for exclusion. This workflow has not been published and no protocol was prepared.

### 2.2. Eligibility Criteria

Out of the 407 research items retrieved from the database search after duplicate removal, the criteria used for screening the articles were that: (Scr1) the study concerned E. sea bass, (Scr2) examined fully developed fish, therefore excluding larvae, and (Scr3) reported plasma, or serum cortisol levels with their concentration. At this stage, 330 research items were excluded, and the remaining 77 were subjected to more detailed inspection for eligibility, including only studies that: (Eli1) provided information on the number of animals used in each experimental group, (Eli2) provided information regarding the dispersion of the data, either as Standard Deviation (S.D.) or as Standard Error of the Mean (S.E.M.), (Eli3) used the individuals and not the tanks as experimental units and, therefore, presented the results as an average of individuals and not tanks, and (Eli4) it was possible to attribute the data to control or post-stress conditions. After this stage, 8 research items were excluded ([16,26,27,28,29,30,31,32]; Table S2), and the remaining 69 items were used in the meta-analysis.

### 2.3. Data Extraction

All qualitative information of the studies, such as assay type, rearing system, fish size, and water parameters, were retrieved from the text and tables of the research items. Data from the research items were extracted either from tables or graphs reporting the mean value ± S.D. or S.E.M. In the latter case, the software ImageJ was used for image analysis based on measuring the length of the *y*-axis and the length of the projections of the cortisol mean ± S.D. or S.E.M. In cases where various post-stress time points were presented, the time of the peak response was used. Moreover, when more than one control group was presented and in order not to violate the assumption of independency of data for the meta-analysis by analyzing data from the same research item more than once, the control groups were pooled. All cortisol data were analyzed as ng mL^−1^. The vast majority of the studies reported this measuring unit (62 out of 69 studies) or its derivatives ug/dL, ug/mL and ng/dL (4 out of 69 studies). There were 3 studies that reported the results as nmol L^−1^ (nM), and their data were converted to ng mL^−1^ by multiplying with the conversion factor 0.36245.

### 2.4. Coding of Data

Each study was coded for quantitative and qualitative data. Quantitative data included (1) fish body size, (2) water temperature, (3) dissolved oxygen, (4) water salinity, (5) water pH, and (6) stocking density. The qualitative data included (7) assay type used to measure cortisol, (8) rearing system, (9) anesthesia type, and, (10) in cases of post-stress samplings, total time between the application of stress and sampling, defined as classes (e.g., 0–30 min, 30–60 min etc.).

In cases where the quantitative data were reported as a range, the mean value was calculated. For instance, in the study by Tintos et al. 2006 [33] where the body weight was reported to range between 15–20 g, the weight was recorded as the mean between the two values, i.e., 17.5 g.

### 2.5. Statistical Analysis

All statistical analysis was performed in RStudio [34], using the packages “meta” [35], “dmetar” [36], and “tidyverse” [37]. Since considerable between-study heterogeneity was expected, a random-effects model was used to pool effect sizes. The heterogeneity variance τ^2^ was calculated using the restricted maximum likelihood estimator, while the confidence interval around the pooled effect was calculated using the Knapp–Hartung adjustments [38].

Specifically, for the basal and post-stress analysis, the pre-calculated effect sizes were analyzed under the “metagen” function, while analysis of the standardized mean differences (SMD) and their 95% confidence intervals (CIs) between basal and post-stress levels were analyzed under the “metacont” function, using Hedges method to calculate the SMD due to the small number of subjects in most studies [35,39]. Subgroup analysis for the qualitative treatment data was performed using the “byvar” argument and was based on calculating different τ^2^ for each subgroup, and subgroups were tested for significant differences using the Q test. Meta-regression on the quantitative influence data was performed using the “metareg” function and risk of bias was assessed by Egger’s test using the “metabias” function. The forest plot was created using the “forest” function.

## 3. Results

### 3.1. Study Characteristics

The final outcome of the literature search was 69 peer-reviewed studies that concerned circulating cortisol levels in E. sea bass and provided sufficient information on the number of animals used and the population mean values and dispersion (Figure 1). Out of these, 35 studies reported only basal, 12 only post-stress, and 22 both basal and post-stress cortisol concentrations, thus making a total of 57 studies reporting basal levels and 34 studies reporting post-stress levels. Summary characteristics for the research items included in the meta-analysis are presented (Table 2). All 69 studies had reported the weight of the fish.

### 3.2. Basal Levels

**Figure 2:**
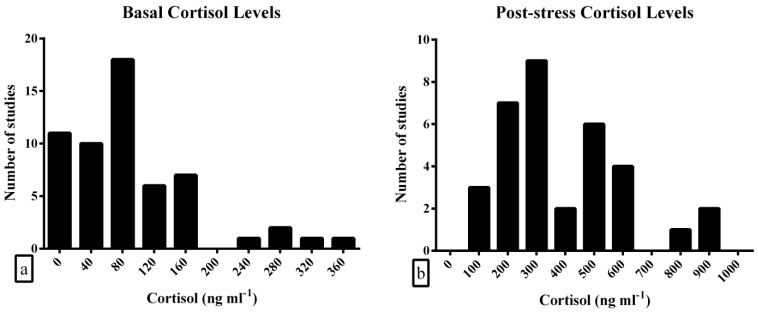
Frequency distribution of meta-analysis data for (**a**) basal and (**b**) post-stress cortisol levels.

Frequency distribution analysis on the basal cortisol levels resulted in a positively skewed distribution (Figure 2a). The pooled effect size was calculated at 88.7 ng mL^−1^ [95%-CI: 65.5–109.8], with a high between-study heterogeneity (Table 3). Egger’s test showed that no significant publication bias was present in the dataset (intercept = 13.5, df = 55; *p* = 0.525).

Subgroup analysis based on the assay type revealed differences between groups. Due to the low number of studies using HPLC and chemiluminescence/electrochemiluminescence assays, an analysis including only the studies using ELISA and RIA assays was performed and showed that the difference between them was significant (Q_1_ = 5.36; *p* = 0.021), being higher in ELISA than RIA. The use of anesthetic also had a significant effect on cortisol (Q_5_ = 12.69; *p* = 0.026), while no differences were observed between the rearing unit systems (Q_3_ = 1.41; *p* = 0.703).

Meta-regression analysis between effect sizes and quantitative characteristics showed that none of the examined parameters, i.e., fish body weight, water temperature, dissolved oxygen concentration, salinity, pH, and stocking density, affected the results.

### 3.3. Post-Stress Levels

Frequency distribution analysis on the post-stress cortisol levels resulted in a positively skewed distribution (Figure 2b). The pooled effect size was calculated at 385.9 ng mL^−1^ [95%-CI: 310.8–460.9], with a high between-study heterogeneity (Table 4). Egger’s test resulted in a marginally significant publication bias in the dataset (intercept = 6.4, df = 32; *p* = 0.047).

Subgroup analysis based on the assay type revealed differences between groups (Q_1_ = 7.76; *p* = 0.005), being higher in ELISA than RIA. The use of anesthetic also had a significant effect on cortisol (Q_3_ = 17.13; *p* < 0.001), excluding the “none”, “ice/cold water”, and “decap/blow to head” groups from the analysis due to the small number of studies in each group. On the other hand, no differences were observed between rearing unit systems (Q_2_ = 5.36; *p* = 0.069), excluding the ponds due to their small number. Time after stress, excluding the “>240” group due to the low number of studies, showed a significant effect (Q_4_ = 9.99; *p* = 0.041). Finally, none of the quantitative parameters was related to the effect sizes when the respective meta-regression analysis was performed.

### 3.4. Standardized Mean Difference between Basal and Post-Stress Cortisol

To assess the difference between basal and post-stress cortisol, 22 studies that included both pre- and post- exposure to acute stress data were used. The pooled SMD was calculated to be 3.02 [95%-CI: 2.46–3.58] (Table 5). The between-study heterogeneity was lower than the ones in the previous analysis, but still significantly large (I^2^ = 81.7%, τ^2^ = 1.12). Egger’s test using the Pustejovsky and Rodgers modification to avoid false positive results that arise with the classical Egger’s test on SMDs [94], resulted in a significant publication bias in the dataset (intercept = 4.7, df = 20; *p* = 0.010).

**Figure 3:**
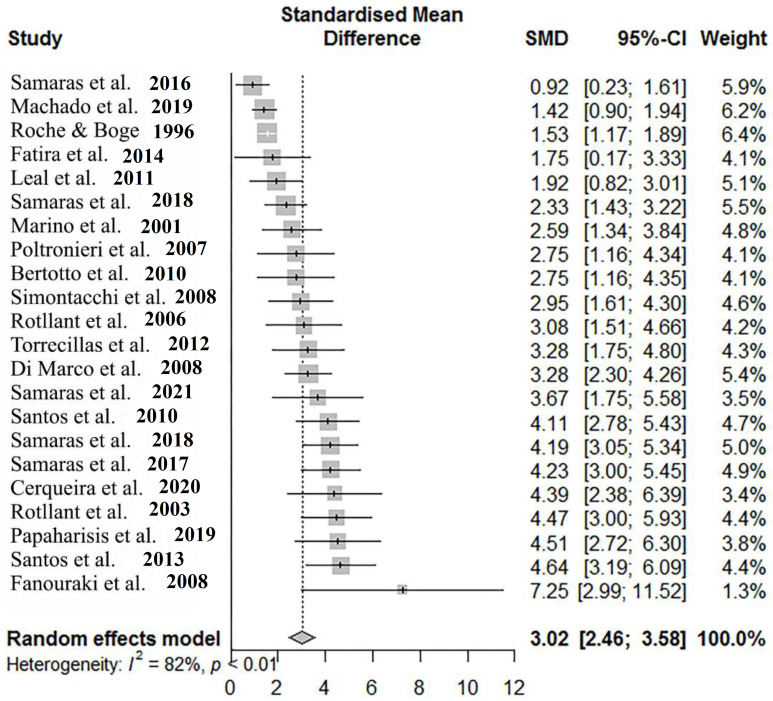
Forest plot depicting the SMD, 95%-CI, and weight of each study included in the analysis. Numbers in the brackets indicate the citation number of each study in the references section. Studies used in this meta-analysis are cited in the references [5,7,10,14,44,54,56,58,61,62,65,68,71,72,74,75,76,77,79,80,82,86].

It is obvious that stress had an overall effect on cortisol, a result that was observed in every study (Figure 3). The high heterogeneity between the studies can also be observed (Figure 3).

Subgroup analysis based on the assay type revealed no significant differences between groups (Q_1_ = 0.03; *p* = 0.870), being similar between studies using ELISA rather than RIA assays. No differences were observed between rearing unit systems, excluding ponds due to the low number of studies in this group (Q_2_ = 0.11; *p* = 0.945). The same was true for the use of anesthetics, analyzing only phenoxyethanol and MS222 (Q_1_ = 0.65; *p* = 0.421). Finally, time after stress had no significant effect on cortisol response (Q_2_ = 1.56; *p* = 0.458) excluding the “0–30”, “120–240”, and “<240” due to low number of studies. Finally, meta-regression analysis between effect sizes and quantitative characteristics showed that none of the examined parameters, i.e., fish body weight, water temperature, dissolved oxygen concentration, salinity, pH, and stocking density, affected the results.

## 4. Discussion

The study of circulating cortisol concentration in E. sea bass is intriguing due to the fact that this species shows high basal and post-stress levels of cortisol, as well as high variation both within the same population and between different studies [4,6,8]. In fact, out of the studied teleost species, E. sea bass is among the ones with the highest reported cortisol levels, together with the chub, *Leuciscus cephalus*, the latter having been characterized as a cortisol resistant species [95]. This high stress susceptibility has been suggested to be co-responsible for disease outbreaks in this species [96].

In this context, a systemic review of the published cortisol levels of E. sea bass could assist in better understanding whether cortisol levels in this species are indeed high as well as to define possible sources of variation between studies. This meta-analysis led to the conclusion that a high between-studies heterogeneity exists in both basal and post-stress concentrations. The reported basal concentrations were calculated to have a pooled effect size of 88.7 ng mL^−1^ with an 95% confidence interval between 65.5–109.8 ng mL^−1^, while post-stress concentration had a pooled effect size of 385.9 ng mL^−1^ [95%-CI: 310.8–460.9].

Subgroup meta-analysis revealed some interesting findings. However, these findings should be interpreted with care since there are constraints in the use of subgroups meta-analyses. The most important ones are the small number of studies in a subgroup and the high between-study heterogeneity, since both reduce the statistical power of the analysis. In order to reduce the “small number of studies” effect in the current analyses, subgroups with lower than three studies were excluded from subgroup meta-analysis. On the other hand, although high heterogeneity was observed in the current study, there are no available tools to mitigate its effect on the subgroups analysis.

Having the above constrains in mind, one of the major factors that seemed to affect the heterogeneity was the assay type. In both basal and post-stress levels, the pooled effect size of studies using ELISA assays was significantly higher than studies using RIA assays. Ιt is generally accepted that RIA has a higher efficiency in measuring cortisol compared to ELISA. In many studies, RIA assays are considered as more accurate when it comes to the analysis of fish cortisol [97], human salivary cortisol [98], as well as mice [99] and bird [100] corticosterone. However, there are also studies in mammals that show equal results between ELISA and RIA [101], or even better performance in the ELISA assays [102]. Therefore, it is difficult to definitely conclude which assay type is more accurate in reporting cortisol levels, but the current study supports the notion that the cortisol assay type should be taken into careful consideration when designing a study and when interpreting the results. On the other hand, when the standardized mean differences between basal and post-stress cortisol levels were analyzed, no difference between assay types was observed. This result indicates that although ELISA assays tend to over-estimate cortisol levels, they do so in a similar manner in basal and post-stress concentrations. In other words, both assay types record the magnitude of the response in the same way although ELISA overestimates the absolute values.

The rearing system, on the other hand, seemed not to affect cortisol levels. The most commonly used systems were the open-flow and the RAS, consisting of approximately 3/4 of the total number of studies. To the best of our knowledge, there are no published studies in E. sea bass to directly compare fish welfare between these rearing systems, though the effects of increased stocking density seem to be the same in fish reared in RAS and open flow systems [55,78]. What has been shown to affect welfare in this species is the size of the rearing unit, in either open flow tanks in larval stages [103] or sea cages during on-growing [76].

Regarding means of anesthesia, most studies used chemical anesthetics, mainly phenoxyethanol, followed by MS222. In both basal and post-stress conditions anesthesia treatment significantly affected the results, showing lower values in decapitated or percussively blown-in-the-head fish. It is well known that E. sea bass is a species with a rapid cortisol response [75], and, therefore, immediate killing does not allow cortisol to rise. In that way, minimum cortisol levels are reported when using this method. In basal conditions, the highest levels were reported in fish that were not under anesthesia, i.e., they were conscious, during blood sampling. When anesthetics were used, phenoxyethanol and MS222 resulted in lower levels than the other anesthetics indicating that, when E. sea bass is sampled for cortisol levels under anesthesia, it is preferable to use one of the aforementioned anesthetics. However, it should be noted that studies assessing direct comparisons between conscious and chemically anesthetized fish, using either phenoxyethanol or clove-oil [59] and MS222 [65], have not reported such differences.

In terms of magnitude of the stress response in relation to the time after stress, it is known that, in E. sea bass, cortisol starts to rise at least 6 min after the application of stress [75], reaching maximum levels at 60 to 120 min post-stress when recovery starts to take place [4,7,14]. Grouping of studies based on the post-stress time at which fish were sampled revealed a similar outcome although it should be noted that the differences were not significant. However, this synthesis of results reflected the typical, more-or less, time-course cortisol response of this species [4,5,14,75], even though different stressors, in terms of nature, intensity, and duration were used.

It is acknowledged that there are some limitations in the conclusions of the current meta-analysis. As mentioned before, the first is due to the high between-studies heterogeneity. This is a result of various reasons, including the different aims of the studies, the different assays used, the rearing methods, temperature, and so on. The second lies to the fact that E. sea bass responds very fast to handling [75], and it is, therefore, difficult to ascertain that the reported basal levels have been obtained under similar sampling stress between studies. This is similar to the source of variation #4 proposed by Ellis et al. [6] (Table 1). Third, there was a scarcity of information in environmental data that could affect the cortisol response, such as water temperature, salinity, pH, and dissolved oxygen concentration. Finally, the circadian rhythm [7,19] the seasonality [5,18], and the sex of the fish [104] are additional potential sources of variation in cortisol levels which are hardly taken into consideration—and subsequently not reported—in most published studies, and, therefore, the current study could not include them in the analysis.

## 5. Conclusions

In conclusion, taking into consideration the limitations discussed above, the current meta-analysis examined 69 studies and calculated a pooled effect for basal and post-stress cortisol levels for E. sea bass. A high between-studies heterogeneity was recorded, with the factors assay type and anesthesia affecting cortisol levels and adding variance to the results. Moreover, a significant effect of acute stress on cortisol levels was observed in all studies examined. On the contrary, no association between cortisol and fish body weight or environmental conditions such as water temperature, dissolved oxygen, salinity pH, stocking density, or the rearing unit was observed. Finally, although it was not possible to directly test for genetic differences between fish, seasonality, circadian rhythms, and sex due to the lack of data in the published studies, the high between-study heterogeneity indicates that these factors may be additional factors causing variation between studies in the examined species.

## Figures and Tables

**Table 1:** Suggested sources of variation in cortisol levels of E. sea bass presented by Ellis et al. (2012) [6], and the respective factors that have been studied thereinafter.

Source of Variation	Reference
1. true differences in basal cortisol concentrations associated with different fish conditions (e.g., body size, age, etc.)	Body size [7]
2. Genetic/strain differences in cortisol responsiveness coping style/personality/temperament/behavioural syndromes	Genetics [8,9,10,11] Coping styles [12,13]
3. different environmental conditions (e.g., water temperature, salinity, lightning, photoperiod, season, food composition)	Temperature [14,15] Salinity [16,17] Photoperiod, season [18,19] Food composition [20,21,22,23]
4. presence of unrecognized stressors (e.g., poor husbandry conditions, disease) in some studies	-
5. the analytical method used (RIA, EIA/ELISA, HPLC) and possible errors	ELISA vs. LC-MS/MS [24]

**Table 2:** Studies included in the meta-analysis (*n* = 69). In Dissolved oxygen, Salinity, pH, and Stocking density a “+” symbol signifies the presence, while a “−“ symbol the absence of information in the respective study.

Study	Year	Treatment	Temperature (°C)	Dissolved Oxygen (mg L^−1^)	Salinity	pH	Stocking Density	Assay Type	Rearing System	Anesthetic	Total Time (min)
[5]	2016	Basal/Post-stress	15.0	−	−	−	+	ELISA	Cage	Phenoxyethanol	30–60
[7]	2014	Basal/Post-stress	-	+	+	−	+	ELISA	Open flow	Clove oil	60–90
[10]	2021	Basal/Post-stress	19.0	−	+	−	+	ELISA	Open flow	Phenoxyethanol	60–90
[11]	2020	Post-stress	18.0	−	+	−	+	ELISA	Open flow	Phenoxyethanol	30–60
[12]	2015	Post-stress	20.1	−	+	−	−	ELISA	RAS	Benzocaine	30–60
[13]	2020	Post-stress	-	−	−	−	−	ELISA	Open flow	Benzocaine	30–60
[14]	2018	Basal/Post-stress	-	−	+	+	+	ELISA	RAS	Phenoxyethanol	60–90
[15]	2020	Basal	16.0	−	+	−	−	ELISA	RAS	MS222	
[18]	2011	Basal	-	−	−	−	−	RIA	Pond	Ice slurry/cold water	
[20]	2013	Post-stress	21.2	+	+	−	−	RIA	RAS	Clove oil	
[21]	2020	Basal	18.0	+	−	+	+	HPLC	RAS	Clove oil	
[22]	2020	Post-stress	22.0	+	−	−	−		Open flow	Phenoxyethanol	90–120
[23]	2017	Basal	25.0	−	+	−	−	ELISA	RAS	Phenoxyethanol	
[24]	2021	Post-stress	21.0	−	−	−	−	ELISA	RAS	Benzocaine	60–90
[33]	2006	Basal	20.0	−	+	−	−	ELISA	Open flow	Phenoxyethanol	
[40]	2009	Basal	19.5	−	−	−	−	RIA	Open flow	-	
[41]	2019	Post-stress	20.9	−	+	+	−	ELISA	RAS	Benzocaine	
[42]	2020	Post-stress	21.5	−	+	+	−	ELISA	RAS	Benzocaine	30–60
[43]	2020	Basal	18.0	−	+	+	+	Chemiluminescence	Open flow	Clove oil	
[44]	2010	Basal/Post-stress	-	−	−	−	−	RIA	Pond	MS222	120–240
[45]	2018	Basal	20.5	+	−	−	−	RIA	-	None	
[46]	2021	Basal	17.5	−	−	−	+	ELISA	RAS	MS222	
[47]	2021	Basal	17.0	−	+	−	+	RIA	RAS	MS222	
[48]	2010	Basal	18.0	−	−	−	+	HPLC	Open flow	Clove oil	
[49]	2014	Basal	18.0	−	+	−	+	HPLC	-	Clove oil	
[50]	2019	Basal	18.0	−	+	−	−	ELISA	RAS	Clove oil	
[51]	2011	Basal	20.0	−	+	+	+	ELISA	Open flow	MS222	
[52]	2012	Basal	18.0	−	−	−	−	ELISA	Open flow	MS222	
[53]	1998	Basal	-	−	−	−	−	RIA	Open flow	MS222	
[54]	2020	Basal/Post-stress	21.9	−	+	+	−	ELISA	Open flow	Phenoxyethanol	0–30
[55]	2010	Basal	21.2	−	+	+	+	RIA	Open flow	Phenoxyethanol	
[56]	2008	Basal/Post-stress	18.5	+	+	−	+	RIA	RAS	Phenoxyethanol	60–90
[57]	2021	Post-stress	21.0	+	+	−	−	RIA	RAS	MS222	0–30
[58]	2008	Basal/Post-stress	20.0	+	+	−	+	ELISA	Open flow	Clove oil	60–90
[59]	2012	Basal	-	−	−	−	−	ELISA	Cage	None	
[60]	2007	Basal	27.3	−	−	−	−	ELISA	Open flow	Phenoxyethanol	
[61]	2011	Basal/Post-stress	-	−	−	−	−	ELISA	Open flow	Phenoxyethanol	90–120
[62]	2019	Basal/Post-stress	25.4	−	+	−	−	ELISA	RAS	Blow to head/decapitation	60–90
[63]	2019	Basal	-	+	+	−	−	ELISA	RAS	Phenoxyethanol	
[64]	2011	Basal	-	−	−	−	−	ELISA	Cage	MS222	
[65]	2001	Basal/Post-stress	14.0	−	+	−	+	RIA	Cage	None	0–30
[66]	2014	Post-stress	20.0	−	+	−	+	ELISA	Open flow	Phenoxyethanol	30–60
[67]	2020	Post-stress	15.7	−	+	−	−	ELISA	Open flow	Phenoxyethanol	120–240
[68]	2019	Basal/Post-stress	12.5	−	−	−	−	ELISA	Cage	Blow to head/decapitation	60–90
[69]	2005	Basal	-	−	−	−	−	RIA	-	Clove oil	
[70]	2011	Basal	20.2	+	+	+	+	RIA	Open flow	MS222	
[71]	2007	Basal/Post-stress	-	−	−	−	+	RIA	Cage	MS222	120–240
[72]	1996	Basal/Post-stress	13.0	−	+	−	−	RIA	Open flow	-	>240
[73]	2010	Basal	27.0	+	+	−	−	Electro- chemiluminescence	Open flow	-	
[74]	2006	Basal/Post-stress	18.0	−	−	−	+	RIA	Open flow	Phenoxyethanol	>240
[75]	2003	Basal/Post-stress	23.0	−	+	−	+	RIA	Open flow	Phenoxyethanol	30–60
[76]	2017	Basal/Post-stress	-	+	+	+	+	ELISA	Cage	Phenoxyethanol	30–60
[77]	2018	Basal/Post-stress	19.0	−	−	−	+	RIA	Open flow	Phenoxyethanol	60–90
[78]	2009	Basal	23.4	−	+	+	+	RIA	RAS	Ice slurry/cold water	
[79]	2013	Basal/Post-stress	22.0	+	+	+	+	ELISA	RAS	MS222	30–60
[80]	2010	Basal/Post-stress	22.0	+	+	+	+	ELISA	RAS	MS222	30–60
[81]	2019	Basal	20.0	−	+	+	−	ELISA	RAS	-	
[82]	2008	Basal/Post-stress	24.0	+	+	−	−	RIA	Pond	Ice slurry/cold water	90–120
[83]	2006	Basal	20.0	+	+	+	−	ELISA	RAS	None	
[84]	2004	Basal	20.0	+	+	+	−	ELISA	RAS	None	
[85]	2005	Basal	20.0	−	+	+	+	ELISA	RAS	MS222	
[86]	2012	Basal/Post-stress	23.0	+	−	−	+	RIA	Open flow	None	90–120
[87]	2015	Basal	19.0	−	+	−	−	ELISA	Open flow	Phenoxyethanol	
[88]	2006	Basal	17.0	−	+	−	−	RIA	Open flow	Ice slurry/cold water	
[89]	2006	Basal	12.5	−	+	−	+	RIA	-	Blow to head/decapitation	
[90]	2010	Basal	18.0	−	−	−	−	ELISA	Open flow	MS222	
[91]	2009	Basal	14.0	−	+	−	−	RIA	Open flow	Phenoxyethanol	
[92]	2012	Post-stress	-	−	−	−	−	RIA	-	Ice slurry/cold water	120–240
[93]	2010	Basal	18.0	+	−	+	−	ELISA	-	None	

**Table 3:** Results of the meta-analysis regarding basal cortisol levels, including subgroup analysis. ES: effect size; 95%-CI: 95% confidence intervals; I^2^: Higgin’s and Thompson’s between-study heterogeneity statistic; τ^2^: heterogeneity variance.

	n	ES	95%-CI	I^2^	τ^2^
Whole population	57	88.7	65.5–109.8	100.0%	6235.7
Assay type					
RIA	22	60.8	37.7–83.8	100.0%	2677.7
ELISA	30	108.2	72.9–143.4	100.0%	8787.1
HPLC	3	97.3	−32.5–227.6	98.9%	2696.8
Chemiluminescence Electrochemiluminescence	2	-	-	-	-
Rearing unit					
Open flow	25	81.1	57.6–104.6	100.0%	3168.4
RAS	17	102.9	48.1–157.7	99.9%	11,200.3
Sea cages	7	95.2	0.1–190.3	100.0%	10,575.0
Ponds	3	113.9	−21.6–249.3	99.4%	2955.7
Anesthesia					
Phenoxyethanol	17	71.7	36.9–106.5	99.9%	4509.7
MS222	14	72.2	29.4–115.0	100.0%	5482.7
Clove oil	8	113.9	80.0–147.8	99.3%	1619.8
None	7	142.3	24.5–260.0	100.0%	16,051.0
Ice/Cold water	4	86.2	−19.9–192.3	100.0%	4433.8
Decap/Blow to head	3	25.6	−77.4–128.7	99.9%	1719.6

**Table 4:** Results of the meta-analysis regarding post-stress cortisol levels, including subgroup analysis. ES: effect size; 95%-CI: 95% confidence intervals; I^2^: Higgin’s and Thompson’s between-study heterogeneity statistic; τ^2^: heterogeneity variance.

	n	ES	95%-CI	I^2^	τ^2^
Whole population	34	385.9	310.8–460.9	99.4%	44,598.1
Assay type					
RIA	13	273.8	180.5–367.1	99.6%	21,989.3
ELISA	20	458.6	352.5–564.6	98.9%	49,493.9
Rearing unit					
Open flow	15	382.6	278.8–486.5	99.5%	34,183.3
RAS	11	452.1	261.4–642.8	99.4%	78,819.7
Sea cages	5	269.4	154.6–384.1	90.0%	7570.1
Ponds	2	-	-	-	-
Anesthesia					
Phenoxyethanol	14	308.1	216.9–399.4	99.6%	24,364.3
MS222	5	273.6	50.37–496.8	96.4%	31,117.1
Clove oil	3	521.0	−119.5–1161.4	94.3%	63,010.3
Benzocaine	5	692.1	439.5–944.7	96.7%	39,320.6
None	2	-	-	-	-
Ice/Cold water	2	-	-	-	-
Decap/Blow to head	2	-	-	-	-
Time post-stress (min)					
0–30	3	259.2	125.8–392.5	87.3%	2353.4
30–60	10	400.6	220.7–580.5	99.6%	60,818.3
60–90	9	415.4	247.1–583.7	99.6%	46,929.3
90–120	4	423.1	228.4–617.8	89.7%	12,499.1
120–240	4	267.0	72.9–461.2	97.5%	11,913.2
>240	2	-	-	-	-

**Table 5:** Results of the meta-analysis regarding the standardized mean difference (SMD) between basal and post-stress cortisol levels, including subgroup analysis. ES: effect size; 95%-CI: 95% confidence intervals; I^2^: Higgin’s and Thompson’s between-study heterogeneity statistic; τ^2^: heterogeneity variance.

	n	SMD	95%-CI	I^2^	τ^2^
Whole population	22	3.02	2.46–3.58	81.7%	1.12
Assay type					
RIA	10	3.00	2.36–3.65	79.8%	0.61
ELISA	12	3.09	2.07–4.11	84.4%	1.86
Rearing unit					
Open flow	10	3.16	2.16–4.16	81.9%	1.21
RAS	5	3.04	1.42–4.67	87.5%	1.45
Sea cages	5	2.90	1.06–4.74	87.1%	1.87
Ponds	2	-	-	-	-
Anesthesia					
Phenoxyethanol	10	3.13	2.24–4.02	82.5%	1.25
MS222	4	3.63	2.11–5.15	36.3%	0.32
Clove oil	2	-	-	-	-
None	2	-	-	-	-
Ice/Cold water	1	-	-	-	-
Decap/Blow to head	2	-	-	-	-
Time post-stress (min)					
0–30	2	-	-	-	-
30–60	5	3.59	1.59–5.59	91.7%	2.39
60–90	8	3.09	1.86–4.32	81.9%	1.30
90–120	3	2.60	0.78–4.41	19.8%	0.15
120–240	2	-	-	-	-
>240	2	-	-	-	-

## Data Availability

Data available upon reasonable request.

## Supplementary Materials

The following supporting information can be downloaded at: https://www.mdpi.com/article/10.3390/ani13081340/s1, Table S1: PRISMA 202 Checklist; Table S2: Excluded studies due to violation of exclusion criteria.
