# Supplementary material for: A Systematic Review and Meta-Analysis of Basal and Post-Stress Circulating Cortisol Concentration in an Important Marine Aquaculture Fish Species, European Sea Bass, Dicentrarchus labrax
Source: Animals (Basel). 2023 Apr 13;13(8):1340. doi: 10.3390/ani13081340 (PMC10135258; doi:10.3390/ani13081340)
Supplement: Supplementary file 1 [file animals-13-01340-s001.zip › Table S2.pdf]

Supplementary Table S2. Criteria for excluding studies due to the eligibility criteria.

Excluded study	Exclusion reason
[16]	Not possible to attribute the data to control or post-stress conditions due to experimental design
[26]	Data presented as tank mean
[27]	Lack of information on dispersion data
[28]	No provision of number of animals under study
[29]	Lack of information on dispersion data
[30]	Data on control fish were shared with [93]
[31]	Lack of information on dispersion of data
[32]	Lack of information on dispersion of data
